# Supplementary material for: Translation of SARS-CoV-2 gRNA Is Extremely Efficient and Competitive despite a High Degree of Secondary Structures and the Presence of an uORF
Source: Viruses. 2022 Jul 8;14(7):1505. doi: 10.3390/v14071505 (PMC9322171; doi:10.3390/v14071505)
Supplement: Supplementary file 1 [file viruses-14-01505-s001.zip › viruses-1779316-supplementary.pdf]

*Renilla Reniformis*

M**D**TSKVYDPE... - Globin
M**E**TSKVYDPE... - ORF-1ab
M**F**TSKVYDPE... - ORF-S / ORF-6
M**D**TSKVYDPE... - ORF-3a
M**Y**TSKVYDPE... - ORF-E
M**A**TSKVYDPE... - ORF-M
M**K**TSKVYDPE... - ORF-7a / ORF-8
M**I**TSKVYDPE... - ORF-7b
M**S**TSKVYDPE... - ORF-N

**Figure S1**. N-terminal modification of the Renilla luciferase coding region. Sequence alignment of the beginning of the Renilla coding region from the SARS-CoV-2 5′UTRs construct used in the figure 3. As the nucleotide context of the AUG codon is conserved; this only induced a modification of the second amino acid (indicated in bold).

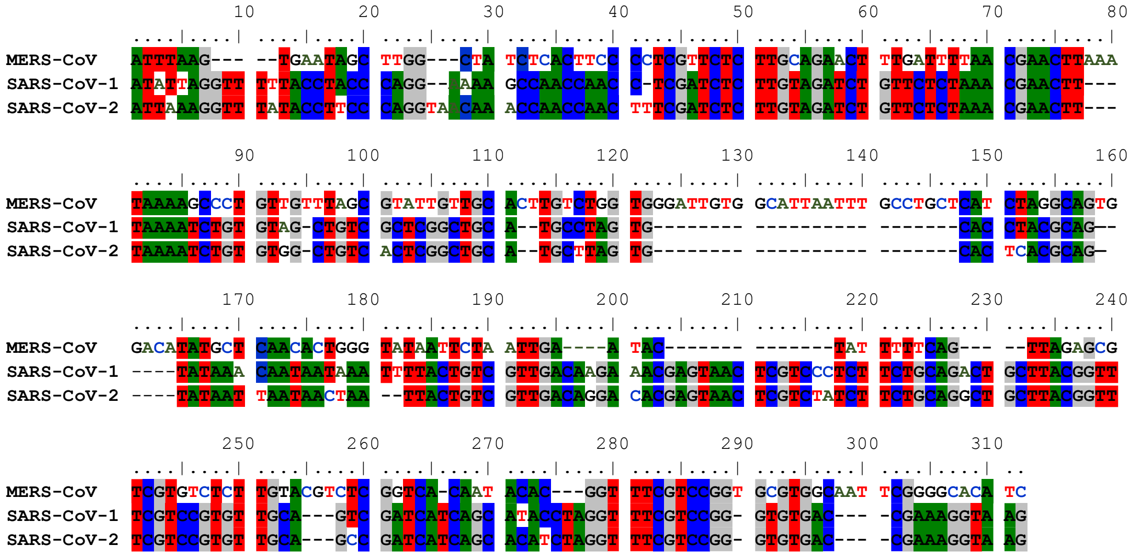

**Figure S2**. SARS CoV-1/2 and MERS CoV gRNA 5′UTRs conservation. The sequences of the 5′UTR from the SARS-CoV type 1 and 2 and MERS-CoV gRNA have been analyzed with a MUltiple Sequence Comparison by Log- Expectation (MUSCLE) using the default parameters. Nucleotide identity has been highlighted in green for adenosine, blue for cytosine, red for tyrosine and grey for guanosine.

**A**

```
                       10
                  ....|....|
SARS-CoV-2 uORF   MLSALTQYN-
SARS-CoV-1 uORF   .P..P...KQ
MERS-CoV   uORF   ..NTGYNS.-
```

**B**

| | MERS-CoV | SARS-CoV-1 |
|---|---|---|
| SARS-CoV-1 | 11,11 | |
| SARS-CoV-2 | 33,33 | 66,67 |

**Figure S3**. 5′UTR$^{ORF1ab}$ uORFs conservation. (**A**) The sequence of the 5′UTR$^{ORF1ab}$ uORFs from SARS-CoV-1, SARS-CoV-2 and MERS-CoV were analyzed with a MUltiple Sequence Comparison by Log- Expectation (MUSCLE) using the default parameters. (**B**) Percentage of identity between the uORFs from 5′UTRs of MERS-CoV, SARS-CoV-1 and SARS-CoV-2 gRNAs resulting from a MUSCLE alignment.

| Constructs | # PCR | 5'-3' Primers | Template |
|---|---|---|---|
| pSARS-CoV-2 Leader_Renilla | PCR #1 | ATCTCTTGTAGATCTGTTCTCTAAACGAACATGGATCCTACTTCGAAAGTTTATG | pGlo-Renilla |
| | | AGATGTAAAGCTTGCATAAATGTTAT | |
| | PCR #2 | TAAGCAGGATCCTAATACGACTCACTATAGATTAAAGGTTTATACCTTCCCAGGTAACAAACCAACCAACTTTCGATCTCTTGTAGATCTGTTCTCTAA | PCR #1 |
| | | AGATGTAAAGCTTGCATAAATGTTAT | |
| | | | |
| pSARS-CoV-2 5'UTR$^{ORF1ab}$ Renilla | PCR #1 | TTCGTCCGTGTTGCAGCCGATCATCAGCACATCTAGGTTTCGTCCGGGTGTGACCGAAAGGTAAGATGGATCCTACTTCGAAAGTTTATG | pGlo-Renilla |
| | | AGATGTAAAGCTTGCATAAATGTTAT | |
| | PCR #2 | AATTAATAACTAATTACTGTCGTTGACAGGACACGAGTAACTCGTCTATCTTCTGCAGGCTGCTTACGGTTTCGTCCGTGTTGCAGCCG | PCR #1 |
| | | AGATGTAAAGCTTGCATAAATGTTAT | |
| | PCR #3 | GTTCTCTAAACGAACTTTAAAATCTGTGTGGCTGTCACTCGGCTGCATGCTTAGTGCACTCACGCAGTATAATTAATAACTAATTACTGTCGTTGACAG | PCR #2 |
| | | AGATGTAAAGCTTGCATAAATGTTAT | |
| | PCR #4 | TAAGCAGAATTCAGATCTATTAAAGGTTTATACCTTCCCAGGTAACAAACCAACCAACTTTCGATCTCTTGTAGATCTGTTCTCTAAACGAACTTTAAAATCTGTG | PCR #3 |
| | | AGATGTAAAGCTTGCATAAATGTTAT | |
| | PCR #5 | TAAGCAGGATCCTAATACGACTCACTATAGGCTAGCATTAAAGGTTTATACCTTCCCAGG | PCR #4 |
| | | AGATGTAAAGCTTGCATAAATGTTAT | |
| | | | |
| pSARS-CoV-1 Leader_Renilla | PCR #1 | ATATTAGGTTTTTACCTACCCAGGAAAAGCCAACCAACCTCGATCTCTTGTAGATCTGTTCTCTAAACGAACATGGATCCTACTTCGAAAGTTTATG | pGlo-Renilla |
| | | AGATGTAAAGCTTGCATAAATGTTAT | |
| | PCR #2 | TAAGCAGGATCCCTCGAGTAATACGACTCACTATAGATATTAGGTTTTTACCTACCCAGG | PCR #1 |
| | | AGATGTAAAGCTTGCATAAATGTTAT | |
| | | | |
| pSARS-CoV-1 5'UTR$^{ORF1ab}$ Renilla | PCR #1 | TGCTTACGGTTTCGTCCGTGTTGCAGTCGATCATCAGCATACCTAGGTTTCGTCCGGGTGTGACCGAAAGGTAAGATGGATCCTACTTCGAAAGTTTATG | pGlo-Renilla |
| | | AGATGTAAAGCTTGCATAAATGTTAT | |
| | PCR #2 | ACCTACGCAGTATAAACAATAATAAATTTTACTGTCGTTGACAAGAAACGAGTAACTCGTCCCTCTTCTGCAGACTGCTTACGGTTTCGTCCGT | PCR #1 |
| | | AGATGTAAAGCTTGCATAAATGTTAT | |
| | PCR #3 | TCGATCTCTTGTAGATCTGTTCTCTAAACGAACTTTAAAATCTGTGTAGCTGTCGCTCGGCTGCATGCCTAGTGCACCTACGCAGTATAAACAATAATAA | PCR #2 |
| | | AGATGTAAAGCTTGCATAAATGTTAT | |
| | PCR #4 | TAAGCAGGATCCCTCGAGTAATACGACTCACTATAGATATTAGGTTTTTACCTACCCAGGAAAAGCCAACCAACCTCGATCTCTTGTAGATCTGTTCT | PCR #3 |
| | | AGATGTAAAGCTTGCATAAATGTTAT | |
| | | | |
| pMERS-CoV Leader_Renilla | PCR #1 | ATTTAAGTGAATAGCTTGGCTATCTCACTTCCCCTCGTTCTCTTGCAGAACTTTGATTTTAACGAACATGGATCCTACTTCGAAAGTTTATG | pGlo-Renilla |
| | | AGATGTAAAGCTTGCATAAATGTTAT | |
| | PCR #2 | TAAGCAGGATCCCTCGAGTAATACGACTCACTATAGATTTAAGTGAATAGCTTGGCTATC | PCR #1 |
| | | AGATGTAAAGCTTGCATAAATGTTAT | |
| | | | |
| pMERS-CoV 5'UTR$^{ORF1ab}$ Renilla | PCR #1 | TAGAGCGTCGTGTCTCTTGTACGTCTCGGTCACAATACACGGTTTCGTCCGGTGCGTGGCAATTCGGGGCACATCATGGATCCTACTTCGAAAGTTTATG | pGlo-Renilla |
| | | AGATGTAAAGCTTGCATAAATGTTAT | |
| | PCR #2 | AATTTGCCTGCTCATCTAGGCAGTGGACATATGCTCAACACTGGGTATAATTCTAATTGAATACTATTTTTCAGTTAGAGCGTCGTGTCTCTTGTA | PCR #1 |
| | | AGATGTAAAGCTTGCATAAATGTTAT | |
| | PCR #3 | TAAAAGCCCTGTTGTTTAGCGTATTGTTGCACTTGTCTGGTGGGATTGTGGCATTAATTTGCCTGCTCATCTAGGC | PCR #2 |
| | | AGATGTAAAGCTTGCATAAATGTTAT | |
| | PCR #4 | TCACTTCCCCTCGTTCTCTTGCAGAACTTTGATTTTAACGAACTTAAATAAAAGCCCTGTTGTTTAGCG | PCR #3 |
| | | AGATGTAAAGCTTGCATAAATGTTAT | |
| | PCR #5 | TACGAAGGATCCTAATACGACTCACTATAGATTTAAGTGAATAGCTTGGCTATCTCACTTCCCCTCGTTCTCTTG | PCR #4 |
| | | AGATGTAAAGCTTGCATAAATGTTAT | |

**Table S1**. List of primers used for the cloning of the SARS-CoV 1/2 and MERS-CoV leader and 5'UTR$^{ORF1ab}$.

| Constructs | # PCR | 5'-3' Primers | Template |
|---|---|---|---|
| pSARS-CoV-2 5'UTR-ORF-1ab Renilla | PCR #1 | TTCGTCCGTGTTGCAGCCGATCATCAGCACATCTAGGTTTCGTCCGGGTGTGACCGAAAGGTAAGATGGAGACTTCGAAAGTTTATGATCCAG | pGlo-Renilla |
| | | TTCTCTGATATCTTCAGTTTGTGTC | |
| | PCR #2 | AATTAATAACTAATTACTGTCGTTGACAGGACACGAGTAACTCGTCTATCTTCTGCAGGCTGCTTACGGTTTCGTCCGTGTTGCAGCCG | PCR #1 |
| | | TTCTCTGATATCTTCAGTTTGTGTC | |
| | PCR #3 | GTTCTCTAAACGAACTTTAAAATCTGTGTGGCTGTCACTCGGCTGCATGCTTAGTGCACTCACGCAGTATAATTAATAACTAATTACTGTCGTTGACAG | PCR #2 |
| | | TTCTCTGATATCTTCAGTTTGTGTC | |
| | PCR #4 | TAAGCAGAATTCAGATCTATTAAAGGTTTATACCTTCCCAGGTAACAAACCAACCAACTTTCGATCTCTTGTAGATCTGTTCTCTAAACGAACTTTAAAATCTGTG | PCR #3 |
| | | TTCTCTGATATCTTCAGTTTGTGTC | |
| | PCR #5 | TAAGCAGGATCCTAATACGACTCACTATAGATTAAAGGTTTATACCTTCCCAGGTAACAAACCAACCAACTTTCGATCTCTTGTAGATCTGTTCTCTAA | PCR #4 |
| | | TTCTCTGATATCTTCAGTTTGTGTC | |
| | | | |
| pSARS-CoV-2 5'UTR-ORF-S Renilla | PCR #1 | TGTAGATCTGTTCTCTAAACGAACAATGTTTACTTCGAAAGTTTATGATCCAG | pGlo-Renilla |
| | | TTCTCTGATATCTTCAGTTTGTGTC | |
| | PCR #2 | TAAGCAGGATCCTAATACGACTCACTATAGATTAAAGGTTTATACCTTCCCAGGTAACAAACCAACCAACTTTCGATCTCTTGTAGATCTGTTCTCTAAACGAAC | PCR #1 |
| | | TTCTCTGATATCTTCAGTTTGTGTC | |
| | | | |
| pSARS-CoV-2 5'UTR-ORF-3a Renilla | PCR #1 | TGTAGATCTGTTCTCTAAACGAACTTATGGATACTTCGAAAGTTTATGATCCAG | pGlo-Renilla |
| | | TTCTCTGATATCTTCAGTTTGTGTC | |
| | PCR #2 | TAAGCAGGATCCTAATACGACTCACTATAGATTAAAGGTTTATACCTTCCCAGGTAACAAACCAACCAACTTTCGATCTCTTGTAGATCTGTTCTCTAAACGAAC | PCR #1 |
| | | TTCTCTGATATCTTCAGTTTGTGTC | |
| | | | |
| pSARS-CoV-2 5'UTR-ORF-E Renilla | PCR #1 | TGTAGATCTGTTCTCTAAACGAACTTATGTACACTTCGAAAGTTTATGATCCAG | pGlo-Renilla |
| | | TTCTCTGATATCTTCAGTTTGTGTC | |
| | PCR #2 | TAAGCAGGATCCTAATACGACTCACTATAGATTAAAGGTTTATACCTTCCCAGGTAACAAACCAACCAACTTTCGATCTCTTGTAGATCTGTTCTCTAAACGAAC | PCR #1 |
| | | TTCTCTGATATCTTCAGTTTGTGTC | |
| | | | |
| pSARS-CoV-2 5'UTR-ORF-M Renilla | PCR #1 | TGTAGATCTGTTCTCTAAACGAACTAAATATTATATTAGTTTTTCTGTTTGGAACTTTAATTTTAGCCATGGCAACTTCGAAAGTTTATGATCCAG | pGlo-Renilla |
| | | TTCTCTGATATCTTCAGTTTGTGTC | |
| | PCR #2 | TAAGCAGGATCCTAATACGACTCACTATAGATTAAAGGTTTATACCTTCCCAGGTAACAAACCAACCAACTTTCGATCTCTTGTAGATCTGTTCTCTAAACGAAC | PCR #1 |
| | | TTCTCTGATATCTTCAGTTTGTGTC | |
| | | | |
| pSARS-CoV-2 5'UTR-ORF-6 Renilla | PCR #1 | GACCATTCCAGTAGCAGTGACAATATTGCTTTGCTTGTACAGTAAGTGACAACAGATGTTTACTTCGAAAGTTTATGATCCAG | pGlo-Renilla |
| | | TTCTCTGATATCTTCAGTTTGTGTC | |
| | PCR #2 | CAGGTGACTCAGGTTTTGCTGCATACAGTCGCTACAGGATTGGCAACTATAAATTAAACACAGACCATTCCAGTAGCAGTG | PCR #1 |
| | | TTCTCTGATATCTTCAGTTTGTGTC | |
| | PCR #3 | TGTAGATCTGTTCTCTAAACGAACGCTTTCTTATTACAAATTGGGAGCTTCGCAGCGTGTAGCAGGTGACTCAGGTTTTGC | PCR #2 |
| | | TTCTCTGATATCTTCAGTTTGTGTC | |
| | PCR #4 | TAAGCAGGATCCTAATACGACTCACTATAGATTAAAGGTTTATACCTTCCCAGGTAACAAACCAACCAACTTTCGATCTCTTGTAGATCTGTTCTCTAAACGAAC | PCR #3 |
| | | TTCTCTGATATCTTCAGTTTGTGTC | |
| | | | |
| pSARS-CoV-2 5'UTR-ORF-7a Renilla | PCR #1 | TGTAGATCTGTTCTCTAAACGAACATGAAAACTTCGAAAGTTTATGATCCAG | pGlo-Renilla |
| | | TTCTCTGATATCTTCAGTTTGTGTC | |
| | PCR #2 | TAAGCAGGATCCTAATACGACTCACTATAGATTAAAGGTTTATACCTTCCCAGGTAACAAACCAACCAACTTTCGATCTCTTGTAGATCTGTTCTCTAAACGAAC | PCR #1 |
| | | TTCTCTGATATCTTCAGTTTGTGTC | |
| | | | |
| pSARS-CoV-2 5'UTR-ORF-7b Renilla | PCR #1 | TTATTGTTGCGGCAATAGTGTTTATAACACTTTGCTTCACACTCAAAAGAAAGACAGAATGATTACTTCGAAAGTTTATGATCCAG | pGlo-Renilla |
| | | TTCTCTGATATCTTCAGTTTGTGTC | |
| | PCR #2 | TGTAGATCTGTTCTCTAAAAGAACTTTACTCTCCAATTTTTCTTATTGTTGCGGCAATAGTG | PCR #1 |
| | | TTCTCTGATATCTTCAGTTTGTGTC | |
| | PCR #3 | TAAGCAGGATCCTAATACGACTCACTATAGATTAAAGGTTTATACCTTCCCAGGTAACAAACCAACCAACTTTCGATCTCTTGTAGATCTGTTCTCTAAACGAAC | PCR #2 |
| | | TTCTCTGATATCTTCAGTTTGTGTC | |
| | | | |
| pSARS-CoV-2 5'UTR-ORF-8 Renilla | PCR #1 | TGTAGATCTGTTCTCTAAACGAACATGAAAACTTCGAAAGTTTATGATCCAG | pGlo-Renilla |
| | | TTCTCTGATATCTTCAGTTTGTGTC | |
| | PCR #2 | TAAGCAGGATCCTAATACGACTCACTATAGATTAAAGGTTTATACCTTCCCAGGTAACAAACCAACCAACTTTCGATCTCTTGTAGATCTGTTCTCTAAACGAAC | PCR #1 |
| | | TTCTCTGATATCTTCAGTTTGTGTC | |
| | | | |
| pSARS-CoV-2 5'UTR-ORF-N Renilla | PCR #1 | TGTAGATCTGTTCTCTAAACGAACAAACTAAAATGTCTACTTCGAAAGTTTATGATCCAG | pGlo-Renilla |
| | | TTCTCTGATATCTTCAGTTTGTGTC | |
| | PCR #2 | TAAGCAGGATCCTAATACGACTCACTATAGATTAAAGGTTTATACCTTCCCAGGTAACAAACCAACCAACTTTCGATCTCTTGTAGATCTGTTCTCTAAACGAAC | PCR #1 |
| | | TTCTCTGATATCTTCAGTTTGTGTC | |

**Table S2.** List of primers used for the cloning of the different 5'UTRs of the SARS-CoV-2 gRNA and sgRNAs

| Constructs | # PCR | 5'-3' Primers | Template |
|---|---|---|---|
| pSARS-CoV-2 5'UTR_Renilla Mut-AUG-uORF | PCR #1.1 | TAAGCAGGATCCCTCGAGTAATACGACTCACTATAGATTAAAGGTTTATACCTTCCCAGGT | pSARS-CoV-2_5'UTR$^{ORF1ab}$_Renilla |
| | | AAGCAGCCGAGTGACAGCCACACAGATTTTAAAGTTCGTTTAGAGAACAGATCTAC | |
| | PCR #1.2 | TGTGTGGCTGTCACTCGGCTGCTTGCTTAGTGCACTCACGCAG | pSARS-CoV-2_5'UTR$^{ORF1ab}$_Renilla |
| | | ACATCAGGATCCATCTTACCTTTCGGTCACACCC | |
| | PCR 2 | TAAGCAGGATCCCTCGAGTAATACGACTCACTATAGATTAAAGGTTTATACCTTCCCAGGT | PCR #1.1 and PCR #1.2 |
| | | ACATCAGGATCCATCTTACCTTTCGGTCACACCC | |
| | | | |
| pSARS-CoV-2 5'UTR_Renilla uORF-phase | PCR #1.1 | TAAGCAGGATCCCTCGAGTAATACGACTCACTATAGATTAAAGGTTTATACCTTCCCAGGT | pSARS-CoV-2_5'UTR$^{ORF1ab}$_Renilla |
| | | GTAATTATATTGTAATTATACTGCGTGAGTGCACTAAGCATG | |
| | PCR #1.2 | AGTATAATTACAATATAATTACTGTCGTTGACAGGACACGAG | pSARS-CoV-2_5'UTR$^{ORF1ab}$_Renilla |
| | | ACATCAGGATCCATCTTTCCTTTCGGACACACCCGGAC | |
| | PCR 2 | TAAGCAGGATCCCTCGAGTAATACGACTCACTATAGATTAAAGGTTTATACCTTCCCAGGT | PCR #1.1 and PCR #1.2 |
| | | ACATCAGGATCCATCTTTCCTTTCGGACACACCCGGAC | |
| | | | |
| pMERS-CoV 5'UTR_Renilla Mut-AUG-uORF | PCR #1 | TCACTTCCCCTCGTTCTCTTGCAGAACTTTGATTTTAACGAACTTAAATAAAAGCCCTGTTGTTTAGCG | pMERS-CoV_5'UTR$^{ORF1ab}$_Renilla |
| | | AGATGTAAAGCTTGCATAAATGTTAT | |
| | PCR #2 | TACGAAGGATCCTAATACGACTCACTATAGATTTAAGTGAATAGCTTGGCTATCTCACTTCCCCTCGTTCTCTTG | PCR #1 |
| | | AGATGTAAAGCTTGCATAAATGTTAT | |
| | PCR #3 | TAAAAGCCCTGTTGTTTAGCGTATTGTTGCACTTGTCTGGTGGGATTGTGGCAATAATTTGCCTGCTCATCTAGGCAGTGGACATTTGCTCAACACTGGG | PCR #2 |
| | | AGATGTAAAGCTTGCATAAATGTTAT | |

**Table S3**. List of primers used for the cloning of the uORFs mutations
